# Supplementary material for: Optical Conductivity as a Probe of the Interaction-Driven Metal in Rhombohedral Trilayer Graphene
Source: Nanomaterials (Basel). 2022 Oct 24;12(21):3727. doi: 10.3390/nano12213727 (PMC9657299; doi:10.3390/nano12213727)
Supplement: Supplementary file 1 [file nanomaterials-12-03727-s001.zip › nanomaterials-1979554-supplementary.pdf]

Supplemental Material for "Optical conductivity as a probe of the interaction-driven metal in rhombohedral trilayer graphene´´

Vladimir Juričić,[1,2] Enrique Muñoz,[3] and Rodrigo Soto-Garrido[3]

[1]Departamento de Física, Universidad Técnica Federico Santa María, Casilla 110, Valparaíso, Chile.
[2]Nordita, KTH Royal Institute of Technology and Stockholm University, Roslagstullsbacken 23, 10691 Stockholm, Sweden
[3]Facultad de Física, Pontificia Universidad Católica de Chile, Vicuña Mackenna 4860, Santiago, Chile

(Dated: November 1, 2022)

CONTENTS

S1. Low-energy model for trilayer graphene 1

S2. Optical conductivity in the Kubo linear-response formalism 1

S3. Paramagnetic metal 2
A. Valence bond order 2
B. Bond current 4
C. Smectic charge-density wave 7
1. Splitting of the band touching points 7
2. Density of states 9
3. Optical conductivity 10

References 11

S1. LOW-ENERGY MODEL FOR TRILAYER GRAPHENE

We consider the low energy model for rhombohedral trilayer graphene (RTG) [1–4]. We use the notation by Szabó and Roy in Ref. [4]. Considering the Nambu (particle-hole), sublattice (or layer), valley and spin degrees of freedom, the single-particle Hamiltonian for noninteracting electrons is given by,

$$H_0 = \alpha\left[f_1(\boldsymbol{k})\Gamma_{3031} + f_2(\boldsymbol{k})\Gamma_{3002}\right] + u\Gamma_{3003} - \mu\Gamma_{3000}, \tag{S1}$$

where $\alpha = t_0^3 a^3/t_\perp$, a is the lattice spacing, $f_1(\boldsymbol{k}) = k_x(k_x^2 - 3k_y^2)$, $f_2(\boldsymbol{k}) = -k_y(k_y^2 - 3k_x^2)$. The form factors $f_1(\mathbf{k})$ and $f_2(\mathbf{k})$ transform under A_{1u} and A_{2u} irreducible representations of D_{3d} point group of the RTG. Momentum $\boldsymbol{k}$ is measured from the respective valleys (band touching points). Electron (hole) doping corresponds to $\mu > 0$ ($\mu < 0$). The sixteen-dimensional matrices are $\Gamma_{\mu\nu\rho\lambda} = \eta_\mu\sigma_\nu\tau_\rho\beta_\lambda$. Four sets of Pauli matrices $\{\eta_\mu\}$, $\{\sigma_\nu\}$, $\{\tau_\rho\}$ and $\{\beta_\lambda\}$ operate on the particle-hole, spin, valley and sublattice indices, respectively, with $\mu, \nu, \rho, \lambda = 0, \cdots, 3$.

S2. OPTICAL CONDUCTIVITY IN THE KUBO LINEAR-RESPONSE FORMALISM

In the Kubo linear-response formalism, the optical conductivity $\sigma_{ij}(\Omega)$ is expressed in terms of the polarization tensor Π_{ij} by the following relation:

$$\sigma_{ij}(\Omega) = \lim_{i\Omega_n \to \Omega + i0^+} \frac{i\Pi_{ij}(i\Omega_n)}{\Omega}, \tag{S2}$$

where the polarization tensor is expressed by the auto-correlation function between the currents

$$\Pi_{ij}(i\Omega_n) = -e^2 T \sum_{n=-\infty}^{\infty} \int \frac{d^2k}{(2\pi)^2} \mathrm{Tr}\left[\hat{v}_i G(i\omega_n + i\Omega_n, \boldsymbol{k})\hat{v}_j G(i\omega_n, \boldsymbol{k})\right]. \tag{S3}$$

Here $\hat{v}_i = \partial H/\partial k_i$, $\omega_n = (2n+1)\pi T$ are the fermionic Matsubara frequencies, and we defiend the (finite temperature) Green´s function for the Hamiltonian Eq.(S1) by

$$G(i\omega_n, \boldsymbol{k}) = \frac{1}{i\omega_n - H} = \frac{1}{(i\omega_n + \mu) - \tilde{H}}, \tag{S4}$$

where $\tilde{H}$ is just $H|_{\mu=0}$.

S3. PARAMAGNETIC METAL

We do not consider superconductivity in our model, and hence the Nambu indices can be omitted. Moreover, we will focus in the paramagnetic case, where the spin indices are trivial. We can therefore write the noninteracting low-energy Hamiltonian as,

$$H_0 = \alpha\left[f_1(\boldsymbol{k})\Gamma_{31} + f_2(\boldsymbol{k})\Gamma_{02}\right] + u\Gamma_{03} - \mu\Gamma_{00}, \tag{S5}$$

where now $\Gamma_{\rho\lambda} = \tau_\rho\beta_\lambda$ and again, $\{\tau_\rho\}$ and $\{\beta_\lambda\}$ are the sets of Pauli matrices that act on the valley and sublattice (layer) indices, respectively. The paramagnetic nature of the metallic state indicates that it is spin-singlet, for which there are three candidates: Valence bond order (A_1), bond current (A_2) and smectic charge-density-wave (E), respectively, represented by the matrices [4]

$$\Gamma_{\rho 1}, \Gamma_{\rho 2}, \text{ and } (\Gamma_{\rho 0}, \Gamma_{\rho 3}),$$

with $\rho = 1, 2$. Their irreducible representations in the D_3 group, which is a subgroup of D_{3d} point group of RTG, are shown inside the parentheses. Here $\rho = 1, 2$ explicitly account for the valley mixing in this phase, which implies the lowering of the symmetry from D_{3d} down to D_3. The smectic charge-density-wave (sCDW) also breaks rotational symmetry about the z direction, generated by Γ_{33}.

A. Valence bond order

In this section we consider the valence bond order (VBO) in our model. The operator representing the VBO is given by:

$$H_{\mathrm{V}BO} = \Delta_1\Gamma_{11} + \Delta_2\Gamma_{21}, \tag{S6}$$

therefore the total Hamiltonian that includes this VBO contribution is:

$$H = H_0 + H_{\mathrm{V}BO}. \tag{S7}$$

In order to calculate the optical conductivity defined by Eq.(S2), we first compute the Green's function from Eq.(S4). For the VBO case, the different terms in $\tilde{H}$ anticommute, so it is straightforward to find

$$G(i\omega_n, \boldsymbol{k}) = \frac{1}{(i\omega_n + \mu) - \tilde{H}} = \frac{(i\omega_n + \mu) + \tilde{H}}{(i\omega_n + \mu)^2 - \tilde{H}^2} = \frac{(i\omega_n + \mu) + \tilde{H}}{(i\omega_n + \mu)^2 - (u^2 + \alpha^2 k^6 + \Delta^2)}, \tag{S8}$$

where we have defined $\Delta^2 = \Delta_1^2 + \Delta_2^2$. To evaluate the polarization tensor Eq. (S3), we also need to compute the velocities $\hat{v}_x$ and $\hat{v}_y$, which are explicitly given by

$$\hat{v}_x = \alpha\left[3(k_x^2 - k_y^2)\Gamma_{31} + 6k_xk_y\Gamma_{02}\right], \quad \hat{v}_y = \alpha\left[-6k_xk_y\Gamma_{31} + 3(k_x^2 - k_y^2)\Gamma_{02}\right]. \tag{S9}$$

We now compute the components $\Pi_{xy}(i\Omega_n)$ and $\Pi_{xx}(i\Omega_n)$ combining Eq.(S8) and Eq.(S9) to obtain

$$\mathrm{Tr}\left[\hat{v}_x G(i\omega_n + i\Omega_n, \boldsymbol{k})\hat{v}_y G(i\omega_n, \boldsymbol{k})\right] = \frac{72\alpha^4 k_x k_y k^8}{\left[(i\omega_n + \mu)^2 - (u^2 + \alpha^2 k^6 + \Delta^2)\right]\left[(i\omega_n + i\Omega_n + \mu)^2 - (u^2 + \alpha^2 k^6 + \Delta^2)\right]} \tag{S10}$$

$$\mathrm{Tr}\left[\hat{v}_x G(i\omega_n + i\Omega_n, \boldsymbol{k})\hat{v}_x G(i\omega_n, \boldsymbol{k})\right] = -\frac{36\alpha^2 k^4\left[u^2 + \alpha^2(k_x^2 - k_y^2)k^4 + \Delta^2 - (i\omega_n + \mu)(i\omega_n + i\Omega_n + \mu)\right]}{\left[(i\omega_n + \mu)^2 - (u^2 + \alpha^2 k^6 + \Delta^2)\right]\left[(i\omega_n + i\Omega_n + \mu)^2 - (u^2 + \alpha^2 k^6 + \Delta^2)\right]} \tag{S11}$$

Notice from the above expressions that $\Pi_{xy}=0$ after integration over the momenta, as expected since time reversal symmetry (TRS) is not broken. Having calculated the trace, we now proceed to do the sum over the Matsubara frequencies and the integral over momenta.

The expression in Eq. (S11), at finite temperature, involves an infnite sum over Matsubara frequencies, that can be performed analytically, to obtain

$$T\sum_{n\in\mathbb{Z}}\mathrm{Tr}\left[\hat{v}_x G(i\omega_n+i\Omega_n,\boldsymbol{k})\hat{v}_x G(i\omega_n,\boldsymbol{k})\right]=\frac{72\alpha^2k^4\left(u^2+\Delta^2+\alpha^2k^4k_x^2\right)}{E(k)\left[4E^2(k)+\Omega_n^2\right]}\sum_{s=\pm}s\,n_F\left[s\,E(k)-\mu\right]. \tag{S12}$$

Here, we defined the dispersion relation

$$E(k)=\sqrt{\alpha^2k^6+u^2+\Delta^2}, \tag{S13}$$

and the Fermi distribution function is

$$n_F(z)=\left(e^{z/T}+1\right)^{-1}. \tag{S14}$$

The analytic continuation to real frequency, given by the prescription $i\Omega_n\to\Omega+i0^+$, allows us to obtain the expression for the polarization tensor

$$\Pi_{xx}(\Omega)=72e^2\alpha^2\int\frac{d^2k}{(2\pi)^2}\frac{k^4\left(u^2+\Delta^2+\alpha^2k^4k_x^2\right)}{E(k)\left[4E^2(k)-(\Omega+i0^+)^2\right]}\left\{n_F\left(E(k)-\mu\right)-n_F\left(-E(k)-\mu\right)\right\}. \tag{S15}$$

Upon performing the angular integral first, and subsequently change the variables $k^5dk=(3\alpha^2)^{-1}EdE$, where the integration domain $k\geq 0$ maps into $E\geq\sqrt{u^2+\Delta^2}$, the expression for the polarization tensor is simplified to

$$\Pi_{xx}(\Omega)=\frac{6e^2}{\pi}\int_{\sqrt{u^2+\Delta^2}}^{\Lambda}dE\,\frac{E^2+u^2+\Delta^2}{\left[4E^2-(\Omega+i0^+)^2\right]}\left\{n_F\left(E-\mu\right)-n_F\left(-E-\mu\right)\right\}, \tag{S16}$$

where we defined an energy cutoff of the order $\Lambda\sim\alpha/a^3$, with a the microscopic lattice constant. Moreover by using the identity

$$\begin{aligned}\frac{1}{4E^2-(\Omega+i0^+)^2}&=\frac{1}{4\Omega}\left[\frac{1}{E-\Omega/2-i0^+}-\frac{1}{E+\Omega/2+i0^+}\right]\\&=\mathcal{P}\frac{1}{4E^2-\Omega^2}+i\frac{\pi}{4\Omega}\left[\delta(E-\Omega/2)+\delta(E+\Omega/2)\right],\end{aligned} \tag{S17}$$

where $\mathcal{P}(z)$ stands for Cauchy's principal value, we have

$$\Pi_{xx}(\Omega)=\Re e\Pi_{xx}(\Omega)+i\Im\Pi_{xx}(\Omega), \tag{S18}$$

with

$$\Re e\Pi_{xx}(\Omega)=\frac{6e^2}{\pi}\mathcal{P}\int_{\sqrt{u^2+\Delta^2}}^{\Lambda}dE\,\frac{E^2+u^2+\Delta^2}{\left[4E^2-\Omega^2\right]}\left\{n_F\left(E-\mu\right)-n_F\left(-E-\mu\right)\right\}, \tag{S19}$$

and

$$\Im\Pi_{xx}(\Omega)=\frac{3e^2}{2|\Omega|}\left(\frac{\Omega^2}{4}+u^2+\Delta^2\right)\left\{n_F(\Omega/2-\mu)-n_F(-\Omega/2-\mu)\right\}. \tag{S20}$$

Based on these results, the real part of the optical conductivity is given by the simple analytical expression

$$\Re e\sigma_{xx}(\Omega)=-\frac{\Im\,\Pi_{xx}(\Omega)}{\Omega}=\frac{3e^2}{2\Omega^2}\left(\frac{\Omega^2}{4}+u^2+\Delta^2\right)\left\{n_F(-|\Omega|/2-\mu)-n_F(|\Omega|/2-\mu)\right\}. \tag{S21}$$

As a final remark, we notice that the density of states (DOS) implied by the dispersion Eq.(S13) is given by the expression

$$N_{VBO}(E)=\int\frac{d^2k}{(2\pi)^2}\delta\left(E(k)-E\right)=\frac{1}{2\pi}\int_0^\infty dk\,k\,\delta(\sqrt{\alpha^2k^6+u^2+\Delta^2}-E)$$

$$= \frac{|E|}{6\pi\alpha^2}\left(\frac{E^2-u^2-\Delta^2}{\alpha^2}\right)^{-2/3}\Theta(E-\sqrt{u^2+\Delta^2}). \tag{S22}$$

A remarkable feature of this expression is that the DOS develops a pole at the minimum of the conduction band $E=\sqrt{u^2+\Delta^2}$, and hence it grows as compared to the gapless case $u=\Delta=0$. This effect is observed in an enhancement of the optical conductivity with the gap, as compared to the universal value $\sigma_0=3e^2/8$, in units $e^2/\hbar$, for gapless case, as can be clearly seen in Eq. (S21). This expression is used to obtain the plot in Fig. 2(a) in the main text.

B. Bond current

In this section we consider the bond current (BC) in our model. The operator representing the BC contribution is given by:

$$H_{\mathrm{BC}} = \Delta_1^{(\mathrm{BC})}\Gamma_{12}+\Delta_2^{(\mathrm{BC})}\Gamma_{22}. \tag{S23}$$

Then the total Hamiltonian used to compute the Green's function from Eq.(S4) is

$$H = H_0 + H_{\mathrm{BC}}. \tag{S24}$$

Notice that in this case the different terms in $\tilde{H}$ do not mutually anticommute.

We now compute $\Pi_{xx}(i\Omega_n)$ from Eq.(S3), using the expressions for the velocities in eq. (S9) and the Green's function $G(i\omega_n,\boldsymbol{k})$, to obtain

$$\mathrm{Tr}\left[\hat{v}_x G(i\omega_n+i\Omega_n,\boldsymbol{k})\hat{v}_x G(i\omega_n,\boldsymbol{k})\right] = 36\alpha^2k^4\frac{A}{B} \tag{S25}$$

where we defined the coefficients

$$\begin{aligned}
A =\ & \Delta_{\mathrm{BC}}^6-\Delta_{\mathrm{BC}}^4\left[\left(z+\frac{i\Omega_n}{2}\right)^2+\frac{3}{4}(i\Omega_n)^2+2E_0^2(k)-3u^2\right]+\Delta_{\mathrm{BC}}^2\left[4\left(E_0^2(k)-u^2\right)\left\{\left(z+\frac{i\Omega_n}{2}\right)^2+\frac{1}{4}\left(E_0^2(k)-5u^2\right)\right\}\right.\\
&\left.-u^4-z\left(z+i\Omega_n\right)\left\{\left(z+\frac{i\Omega_n}{2}\right)^2+\frac{3}{4}(i\Omega_n)^2-2u^2\right\}\right]+\left[z\left(z+i\Omega_n\right)-u^2\right]\left[z^2-E_0^2(k)\right]\left[(z+i\Omega_n)^2-E_0^2(k)\right]\\
&+\left(E_0^2(k)-u^2\right)\cos(2\phi)\left\{\Delta_{\mathrm{BC}}^4+\Delta_{\mathrm{BC}}^2\left[6\left(z+\frac{i\Omega_n}{2}\right)^2-\frac{(i\Omega_n)^2}{2}-2\left(E_0^2(k)+2u^2\right)\right]+\left(z^2-E_0^2(k)\right)\left[(z+i\Omega_n)^2-E_0^2(k)\right]\right\}\\
B =\ & \left[z^2-E_+^2(k)\right]\left[z^2-E_-^2(k)\right]\left[(z+i\Omega_n)^2-E_+^2(k)\right]\left[(z+i\Omega_n)^2-E_-^2(k)\right].
\end{aligned} \tag{S26}$$

Here $\Delta_{\mathrm{BC}}=\sqrt{\left[\Delta_1^{(\mathrm{BC})}\right]^2+\left[\Delta_2^{(\mathrm{BC})}\right]^2}$, $z=i\omega_n+\mu$, and we defined the dispersion relations

$$E_0(k)=\sqrt{\alpha^2k^6+u^2},\quad E_\pm[E_0(k)]=\sqrt{\left(\sqrt{E_0^2(k)-u^2}\pm\Delta_{\mathrm{BC}}\right)^2+u^2}. \tag{S27}$$

Since upon integration over $\mathbf{k}$-space the term proportional to $\cos(2\phi)$ in Eq. (S26) will vanish, we only care about the Matsubara sum of the remaining terms in A. Therefore, we obtain

$$\begin{aligned}
T\sum_{n\in\mathbb{Z}}\mathrm{Tr}\left[\hat{v}_x G(i\omega_n+i\Omega_n,\boldsymbol{k})\hat{v}_x G(i\omega_n,\boldsymbol{k})\right] =\ & 36\alpha^2k^4\left\{\bar{\alpha}[E_0(k)]\left[n_F\left(E_+(k)-\mu\right)-n_F\left(-E_+(k)-\mu\right)\right]\right.\\
&\left.+\bar{\gamma}[E_0(k)]\left[n_F\left(E_-(k)-\mu\right)-n_F\left(-E_-(k)-\mu\right)\right]\right\}.
\end{aligned} \tag{S28}$$

After applying analytic continuation into the real frequency space $i\Omega_n\to\Omega+i0^+$, the coefficients reduce to the following expressions

$$\bar{\alpha}[E_0]=\frac{\alpha[E_0]}{D_2[E_0]},\quad \bar{\gamma}[E_0]=\frac{\gamma[E_0]}{D_1[E_0]}. \tag{S29}$$

In these expressions, we defined

$$\alpha[E_0] = E_0^2\left(4u^2\left(-20\Delta^3+3\Delta\Omega^2-\Omega^2\sqrt{E_0^2-u^2}\right)+\left(4\Delta^2-\Omega^2\right)\left(4\Delta^3-\Delta\Omega^2+(12\Delta^2-\Omega^2)\sqrt{E_0^2-u^2}\right)\right)$$
$$-u^2\left(16\Delta^2u^2\left(\sqrt{E_0^2-u^2}-2\Delta\right)+\left(4\Delta^2-\Omega^2\right)\left(4\Delta^3-\Delta\Omega^2+(12\Delta^2+\Omega^2)\sqrt{E_0^2-u^2}\right)\right)$$
$$+4E_0^4\left(4\Delta^2-\Omega^2\right)\left(3\Delta+\sqrt{E_0^2-u^2}\right),$$
$$\gamma[E_0] = E_0^2\left(4u^2\left(20\Delta^3-3\Delta\Omega^2-\Omega^2\sqrt{E_0^2-u^2}\right)+\left(4\Delta^2-\Omega^2\right)\left(-4\Delta^3+\Delta\Omega^2+(12\Delta^2-\Omega^2)\sqrt{E_0^2-u^2}\right)\right)$$
$$+u^2\left(4\Delta^2-\Omega^2\right)\left(4\Delta^3-\Delta\Omega^2-(12\Delta^2+\Omega^2)\sqrt{E_0^2-u^2}\right)-16\Delta^2u^4\left(2\Delta+\sqrt{E_0^2-u^2}\right)$$
$$+4E_0^4\left(4\Delta^2-\Omega^2\right)\left(\sqrt{E_0^2-u^2}-3\Delta\right),$$
$$D_1[E_0] = -2\sqrt{\Delta\left(\Delta-2\sqrt{E_0^2-u^2}\right)+E_0^2}\sqrt{E_0^2-u^2}\left[\left(\Omega+i0^+\right)^2-(\chi_+[E_0])^2\right]\left[\left(\Omega+i0^+\right)^2-(\phi_+[E_0])^2\right]$$
$$\times\left[\left(\Omega+i0^+\right)^2-(\phi_-[E_0])^2\right],$$
$$D_2[E_0] = -2\sqrt{\Delta\left(\Delta+2\sqrt{E_0^2-u^2}\right)+E_0^2}\sqrt{E_0^2-u^2}\left[\left(\Omega+i0^+\right)^2-(\chi_-[E_0])^2\right]\left[\left(\Omega+i0^+\right)^2-(\phi_+[E_0])^2\right]$$
$$\times\left[\left(\Omega+i0^+\right)^2-(\phi_-[E_0])^2\right], \tag{S30}$$

with the functions given by

$$\chi_\pm[E_0] = 2\sqrt{E_0^2+\Delta^2\pm2\Delta\sqrt{E_0^2-u^2}} \tag{S31}$$
$$\phi_\pm[E_0] = \sqrt{2\left(E_0^2+\Delta^2\right)\pm2\sqrt{\left(E_0^2-\Delta^2\right)^2+4u^2\Delta^2}}$$

By applying an analogous decomposition for $D[E_0]$ in terms of its poles, as in Eq. (S17), and inserting into Eq. (S28), we can integrate by the change of variables $E_0dE_0 = 3\alpha^2k^5dk$, where the integration domain $k\geq 0$ maps into $E_0\geq u$. This procedure leads to the analytical expressions

$$\Pi_{xx}(\Omega) = \Re e\Pi_{xx}(\Omega)+i\Im\Pi_{xx}(\Omega), \tag{S32}$$

with

$$\Re e\Pi_{xx}(\Omega) = \frac{3e^2}{\pi}\mathcal{P}\int_u^\Lambda dE_0\frac{E_0}{\sqrt{E_0^2-u^2}\left[\Omega^2-(\phi_+[E_0])^2\right]\left[\Omega^2-(\phi_-[E_0])^2\right]}$$
$$\times\left\{\frac{\alpha[E_0]\left[n_F(E_+-\mu)-n_F(-E_+-\mu)\right]}{\sqrt{\Delta\left(\Delta+2\sqrt{E_0^2-u^2}\right)+E_0^2}\left[\Omega^2-(\chi_-[E_0])^2\right]}+\frac{\gamma[E_0]\left[n_F(E_--\mu)-n_F(-E_--\mu)\right]}{\sqrt{\Delta\left(\Delta-2\sqrt{E_0^2-u^2}\right)+E_0^2}\left[\Omega^2-(\chi_+[E_0])^2\right]}\right\}$$
$$\Im\Pi_{xx}(\Omega) = -3e^2\int_u^\Lambda dE_0\frac{E_0}{\sqrt{E_0^2-u^2}}\left\{\frac{\alpha[E_0]\left[n_F(E_+-\mu)-n_F(-E_+-\mu)\right]}{\sqrt{\Delta\left(\Delta+2\sqrt{E_0^2-u^2}\right)+E_0^2}}\left[\frac{\delta\left(\Omega^2-(\chi_-[E_0])^2\right)}{\left[\Omega^2-(\phi_+[E_0])^2\right]\left[\Omega^2-(\phi_-[E_0])^2\right]}\right.\right.$$
$$\left.+\frac{\delta\left(\Omega^2-(\phi_+[E_0])^2\right)}{\left[\Omega^2-(\chi_-[E_0])^2\right]\left[\Omega^2-(\phi_-[E_0])^2\right]}\right]+\frac{\gamma[E_0]\left[n_F(E_--\mu)-n_F(-E_--\mu)\right]}{\sqrt{\Delta\left(\Delta-2\sqrt{E_0^2-u^2}\right)+E_0^2}}\left[\frac{\delta\left(\Omega^2-(\chi_+[E_0])^2\right)}{\left[\Omega^2-(\phi_+[E_0])^2\right]\left[\Omega^2-(\phi_-[E_0])^2\right]}\right.$$
$$\left.\left.+\frac{\delta\left(\Omega^2-(\phi_+[E_0])^2\right)}{\left[\Omega^2-(\chi_-[E_0])^2\right]\left[\Omega^2-(\phi_-[E_0])^2\right]}\right]\right\}. \tag{S33}$$

By analyzing the support of the delta functions in parameter space, we obtain the expressions

$$\delta\left(\Omega^2-(\chi_+[E_0])^2\right)=\Theta(|\Omega|-2u)\Theta(2\sqrt{(u^2+\Delta^2)}-|\Omega|)\left[\frac{\delta(E_0-\epsilon_1)}{2|\Omega||\mathcal{J}_+(\epsilon_1)|}+\frac{\delta(E_0-\epsilon_2)}{2|\Omega||\mathcal{J}_+(\epsilon_2)|}\right], \tag{S34}$$

$$\delta\left(\Omega^2-(\chi_-[E_0])^2\right)=\frac{\delta(E_0-\epsilon_2)}{2|\Omega||\mathcal{J}_-(\epsilon_2)|}\Theta(|\Omega|-2\sqrt{u^2+\Delta^2}) \tag{S35}$$

$$\delta\left(\Omega^2-(\phi_+[E_0])^2\right)=\frac{\delta(E_0-\epsilon_3)}{2|\Omega||J_+(\epsilon_3)|}\Theta(|\Omega|-2\sqrt{u^2+\Delta^2}), \tag{S36}$$

where we defined the frequency-dependent parameters

$$\epsilon_1=\sqrt{u^2+\left(\Delta+\sqrt{\frac{\Omega^2}{4}-u^2}\right)^2},$$

$$\epsilon_2=\sqrt{u^2+\left(\Delta-\sqrt{\frac{\Omega^2}{4}-u^2}\right)^2},$$

$$\epsilon_3=\sqrt{\frac{u^2\Delta^2}{\Delta^2-\Omega^2/4}+\frac{\Omega^2}{4}}. \tag{S37}$$

Performing the integrals in Eq.(S33), and using the conditions in Eq.(S36), we obtain an explicit analytical expression for the real part of the optical conductivity

$$\Re e\sigma_{xx}(\Omega)=\frac{3e^2}{2\Omega^2}\Bigg[\Theta\left(|\Omega|-2u\right)\Theta\left(2\sqrt{u^2+\Delta^2}-|\Omega|\right)\sum_{j=1,2}\frac{\alpha_-(\epsilon_j)\,\delta n_F(E_-(\epsilon_j))}{|\mathcal{J}_-(\epsilon_j)|\left[\Omega^2-\phi_+^2(\epsilon_j)\right]\left[\Omega^2-\phi_-^2(\epsilon_j)\right]} \tag{S38}$$

$$+\Theta\left(|\Omega|-2\sqrt{u^2+\Delta^2}\right)\left\{\sum_{s=\pm}\frac{\alpha_s(\epsilon_3)\delta n_F(E_s(\epsilon_3))}{|J_+(\epsilon_3)|\left[\Omega^2-\chi_s^2(\epsilon_3)\right]\left[\Omega^2-\phi_-^2(\epsilon_3)\right]}+\frac{\alpha_+(\epsilon_2)\,\delta n_F(E_+(\epsilon_2))}{|\mathcal{J}_+(\epsilon_2)|\left[\Omega^2-\phi_+^2(\epsilon_2)\right]\left[\Omega^2-\phi_-^2(\epsilon_2)\right]}\right\}\Bigg].$$

Here, we defined

$$\delta n_F(E)=n_F(-E-\mu)-n_F(E-\mu), \tag{S39}$$

and the coefficients

$$\alpha_+[\epsilon]=\frac{\epsilon\,\alpha[\epsilon]}{\sqrt{\Delta\left(\Delta+2\sqrt{\epsilon^2-u^2}\right)+\epsilon^2}\sqrt{\epsilon^2-u^2}},$$

$$\alpha_-[\epsilon]=\frac{\epsilon\,\gamma[\epsilon]}{\sqrt{\Delta\left(\Delta-2\sqrt{\epsilon^2-u^2}\right)+\epsilon^2}\sqrt{\epsilon^2-u^2}}, \tag{S40}$$

along with the Jacobian functions

$$\mathcal{J}_\pm[\epsilon]=\frac{\partial\chi_\pm}{\partial\epsilon}=\frac{2\epsilon\left(1\pm\frac{\Delta}{\sqrt{\epsilon^2-u^2}}\right)}{\sqrt{\epsilon^2+\Delta^2\pm2\Delta\sqrt{\epsilon^2-u^2}}}, \tag{S41}$$

$$J_\pm[\epsilon]=\frac{\partial\phi_\pm}{\partial\epsilon}=\frac{2\epsilon\left(1\pm\frac{(\epsilon^2-\Delta^2)}{\sqrt{(\epsilon^2-\Delta^2)^2+4u^2\Delta^2}}\right)}{\sqrt{2\left(\epsilon^2+\Delta^2\right)\pm2\sqrt{\left(\epsilon^2-\Delta^2\right)^2+4u^2\Delta^2}}}. \tag{S42}$$

Eq. (S39) is reduced to a more compact notation, which is shown in the main text, as follows

$$\Re e\sigma_{xx}(\Omega)=\frac{3e^2}{2\Omega^2}\Bigg[\Theta\left(|\Omega|-2u\right)\Theta\left(2\sqrt{u^2+\Delta^2}-|\Omega|\right)\sum_{j=1,2}\mathcal{F}_-[\epsilon_j]\delta n_F(E_-(\epsilon_j))+\Theta\left(|\Omega|-2\sqrt{u^2+\Delta^2}\right)$$

$$\times\left\{\sum_{s=\pm}\mathcal{G}_s[\epsilon_3]\delta n_F(E_s(\epsilon_3))+\mathcal{F}_+[\epsilon_2]\delta n_F(E_+(\epsilon_2))\right\}\Bigg], \tag{S43}$$

where we defined the functions

$$\mathcal{F}_{\pm}[\epsilon] = \frac{\alpha_{\pm}(\epsilon)}{|\mathcal{J}_{\pm}(\epsilon)|\left[\Omega^2 - \phi_{+}^2(\epsilon)\right]\left[\Omega^2 - \phi_{-}^2(\epsilon)\right]},$$
$$\mathcal{G}_{\pm}[\epsilon] = \frac{\alpha_{\pm}(\epsilon)}{|J_{+}(\epsilon)|\left[\Omega^2 - \chi_{\pm}^2(\epsilon)\right]\left[\Omega^2 - \phi_{-}^2(\epsilon)\right]}. \tag{S44}$$

We now consider the DOS from the dispersion relation Eq. (S27). The explicit analytical expression then reads

$$N_{BC}(E) = \int \frac{d^2k}{(2\pi)^2} \sum_{s=\pm,\lambda=\pm} \delta\left(\lambda\sqrt{\left(\alpha k^3 + s\Delta\right)^2 + u^2} - E\right) \tag{S45}$$
$$= \frac{|E|}{6\pi\alpha^{2/3}} \frac{1}{\sqrt{E^2 - u^2}} \left[\left(\sqrt{E^2 - u^2} - \Delta\right)^{-\frac{1}{3}} \Theta(E^2 - u^2 - \Delta^2) + \left(\sqrt{E^2 - u^2} + \Delta\right)^{-\frac{1}{3}} \Theta(E^2 - u^2)\right].$$

This expression is used for the plot in Fig. 2(b).

C. Smectic charge-density wave

In this section, we consider the smectic charge-density wave (sCDW) instability of the RTG. The mean-field Hamiltonian corresponding to the sCDW is given by

$$H_{\rm SCDW} = \Delta_{10}^{\rm (SCDW)}\Gamma_{10} + \Delta_{20}^{\rm (SCDW)}\Gamma_{20} + \Delta_{13}^{\rm (SCDW)}\Gamma_{13} + \Delta_{23}^{\rm (SCDW)}\Gamma_{23}$$
$$\equiv \Delta_0(\cos\chi\Gamma_{10} + \sin\chi\Gamma_{20}) + \Delta_3(\cos\delta\,\Gamma_{13} + \sin\delta\,\Gamma_{23}), \tag{S46}$$

where we now introduced the polar angles χ and δ to parametrize the CDW components $\sim \Gamma_{\rho 0}$ and $\sim \Gamma_{\rho 3}$, with the amplitudes, respectively, given by Δ_0 and Δ_3, both positive. The mean-field band structure, taking $u = 0$ and $\mu = 0$, then reads

$$\tilde{E}_{\lambda,\xi}(\mathbf{k}) = \lambda\sqrt{\Delta_0^2 + \Delta_3^2 + \alpha^2 k^6 + \xi\sqrt{2 f_{\rm SCDW}}}, \tag{S47}$$

with

$$f_{\rm sCDW} = \Delta_0^2\Delta_3^2 + (\Delta_0^2 + \Delta_3^2)\alpha^2 k^6 + \Delta_0^2\Delta_3^2\cos(2\delta - 2\chi)$$
$$+ \alpha^2 k^6\left[(\Delta_3^2 - \Delta_0^2)\cos 6\phi - 2\Delta_0\Delta_3\sin(\delta - \chi)\sin 6\phi\right], \tag{S48}$$

and $\lambda, \xi = \pm$ label four bands.

1. *Splitting of the band touching points*

We now show that that the conduction ($\lambda = +$) and the valence ($\lambda = -$) bands, both with $\xi = -$, touching at zero energy at the two valleys, feature two-triplets of nondegenerate touching points with the linear dispersion. At the touching points, therefore,

$$(\Delta_0^2 + \Delta_3^2 + \alpha^2 k^6)^2 - 2 f_{\rm sCDW} = 0. \tag{S49}$$

Introducing $x = \alpha^2 k^6$, the solution of this equation explicitly reads

$$x_{\pm} = -\left[(\Delta_0^2 - \Delta_3^2)\cos 6\phi + 2\Delta_0\Delta_3\sin(\delta - \chi)\sin 6\phi\right] \pm i|2\Delta_0\Delta_3\sin(\delta - \chi)\cos 6\phi + (\Delta_3^2 - \Delta_0^2)\sin 6\phi|. \tag{S50}$$

To realize the band touching, the imaginary part of the above solution should vanish,

$$2\Delta_0\Delta_3\sin(\delta - \chi)\cos 6\phi + (\Delta_3^2 - \Delta_0^2)\sin 6\phi = 0, \tag{S51}$$

implying that at the band touching point

$$\tan 6\phi_0 = \frac{2\Delta_0\Delta_3\sin(\delta - \chi)}{\Delta_0^2 - \Delta_3^2}. \tag{S52}$$

The polar angle of the band touching point is therefore well defined for any sCDW configuration. For the values of the polar angle in Eq. (S52), the solution for the radial coordinate of the band-touching point in Eq. (S50), takes the form

$$x_0 = -\frac{\sin 6\phi_0}{2\Delta_0\Delta_3\sin(\delta-\chi)}\left[(\Delta_0^2-\Delta_3^2)^2+4\Delta_0^2\Delta_3^2\sin^2(\delta-\chi)\right], \tag{S53}$$

which has to be positive. We now show that this is, indeed, the case, and such a solution for $x_0 = \alpha k_0^6$ implies that there are six angles where the bands touch, i.e. there are six band touching points with the same absolute value of the momentum. We consider four separate cases as follows:

1. $\sin(\delta-\chi) > 0$ & $\Delta_0 - \Delta_3 < 0$ implying that $\sin 6\phi < 0, \cos 6\phi > 0$. According to Eq. (S52), the corresponding solutions for the angle are given by

$$\phi_0 = n\frac{\pi}{3} - \frac{1}{6}\arcsin\frac{2\Delta_0\Delta_3\sin(\delta-\chi)}{\sqrt{(\Delta_0^2-\Delta_3^2)^2+4\Delta_0^2\Delta_3^2\sin^2(\delta-\chi)}}, \tag{S54}$$

with $n = 0,1,2,3,4,5$, with the value of the momentum at the band touching points given by Eq. (S50).

2. $\sin(\delta-\chi) > 0$ & $\Delta_0 - \Delta_3 > 0$ implying that $\sin 6\phi_0 < 0, \cos 6\phi_0 < 0$. Therefore, according to Eq. (S52), the corresponding solutions for the angle are given by

$$\phi_0 = (2n+1)\frac{\pi}{6} + \frac{1}{6}\arcsin\frac{2\Delta_0\Delta_3\sin(\delta-\chi)}{\sqrt{(\Delta_0^2-\Delta_3^2)^2+4\Delta_0^2\Delta_3^2\sin^2(\delta-\chi)}}, \tag{S55}$$

with $n = 0,1,2,3,4,5$, with the value of the momentum at the band touching points given by Eq. (S50).

3. $\sin(\delta-\chi) < 0$ & $\Delta_0 - \Delta_3 < 0$ implying that $\sin 6\phi_0 > 0, \cos 6\phi_0 > 0$. Therefore, according to Eq. (S52), the corresponding solutions for the angle are given by

$$\phi_0 = n\frac{\pi}{3} - \frac{1}{6}\arcsin\frac{2\Delta_0\Delta_3\sin(\delta-\chi)}{\sqrt{(\Delta_0^2-\Delta_3^2)^2+4\Delta_0^2\Delta_3^2\sin^2(\delta-\chi)}}, \tag{S56}$$

with $n = 0,1,2,3,4,5$, with the value of the momentum at the band touching points given by Eq. (S50).

4. $\sin(\delta-\chi) < 0$ & $\Delta_0 - \Delta_3 > 0$ implying that $\sin 6\phi_0 > 0, \cos 6\phi_0 < 0$. Therefore, according to Eq. (S52), the corresponding solutions for the angle are given by

$$\phi_0 = (2n+1)\frac{\pi}{6} + \frac{1}{6}\arcsin\frac{2\Delta_0\Delta_3\sin(\delta-\chi)}{\sqrt{(\Delta_0^2-\Delta_3^2)^2+4\Delta_0^2\Delta_3^2\sin^2(\delta-\chi)}}, \tag{S57}$$

with $n = 0,1,2,3,4,5$, with the value of the momentum at the band touching points given by Eq. (S50).

Finally, the explicit solutions in cases when one of the sCDW components vanishes read:

(a) For $\Delta_0 = 0$, $\Delta_3 \neq 0$,

$$k_0 = \left(\frac{\Delta_3}{\alpha}\right)^{1/3}, \quad \phi_0 = n\frac{\pi}{3}; \tag{S58}$$

(b) For $\Delta_0 \neq 0$, $\Delta_3 = 0$,

$$k_0 = \left(\frac{\Delta_0}{\alpha}\right)^{1/3}, \quad \phi_0 = (2n+1)\frac{\pi}{6}, \tag{S59}$$

with $n = 0,1,2,3,4,5$.

Expansion of the Hamiltonian in Eq. (S46) about these six band touching points shows that they split into two triplets, with the vorticity equal to $\pm\pi$. Therefore, two cubic band touching points at the two valleys, with the net zero vorticity, $0 = 3\pi - 3\pi$, split in the sCDW phase into two triplets of gapless points of the vorticity equal to $\pm\pi$, so that the total vorticity is still vanishing, as it should. Finally, we emphasize that the states of the new band touching points in the sCDW are all admixtures of the low-energy states living in single valleys of the noninteracting TBLG.

2. Density of states

To find the form of the DOS in the sCDW case, we can, therefore, without loss of generality, choose, for instance, $\Delta_3 = 0$. Then the total Hamiltonian involving the sCDW is

$$H = H_0 + H_{\rm sCDW} \tag{S60}$$

where

$$\Delta = \sqrt{\left[\Delta_{10}^{(\rm sCDW)}\right]^2 + \left[\Delta_{20}^{(\rm sCDW)}\right]^2}, \quad z = i\omega_n + \mu.$$

The dispersion relation that follows from this Hamiltonian is, for $\lambda = \pm$ and $\xi = \pm$, respectively,

$$\tilde{E}_{\lambda,\xi}(\mathbf{k}) = \lambda\sqrt{\alpha^2 k^6 + u^2 + \Delta^2 + \xi\Delta\sqrt{2\alpha^2 k^6(1-\cos(6\phi)) + 4u^2}}. \tag{S61}$$

It is then more convenient to rewrite this expression by defining the dispersion, after setting $\mu = 0$,

$$\tilde{E}_0(k) = \sqrt{\alpha^2 k^6 + u^2 + \Delta^2}, \tag{S62}$$

such that, using $2\sin^2(3\phi) = [1-\cos(6\phi)]$,

$$\tilde{E}_{\lambda,\xi}(k, 3\phi) = \lambda\sqrt{\tilde{E}_0^2 + 2\xi\Delta\sqrt{\left(\tilde{E}_0^2 - u^2 - \Delta^2\right)\sin^2(3\phi) + u^2}}. \tag{S63}$$

Let us calculate the density of states, as follows

$$\begin{aligned} N_{sCDW}(E) &= \sum_{\lambda=\pm,\xi=\pm}\int \frac{d^2k}{(2\pi)^2}\delta(E - \tilde{E}_{\lambda,\xi}(k,3\phi)) = \sum_{\lambda=\pm,\xi=\pm}\int_0^\infty \frac{dk\,k}{(2\pi)^2}\int_0^{2\pi} d\phi\delta(E - \tilde{E}_{\lambda,\xi}(k,3\phi)) \\ &= \frac{1}{3}\sum_{\lambda=\pm,\xi=\pm}\int_0^\infty \frac{dk\,k}{(2\pi)^2}\int_0^{6\pi} d\phi\delta(E - \tilde{E}_{\lambda,\xi}(k,\phi) - E), \end{aligned} \tag{S64}$$

where in the last step we made the change of variable in the angular coordinate $3\phi \to \phi$. Now, we split the angular integral into three subintervals, $[0,6\pi] = [0,2\pi]\cup[2\pi,4\pi]\cup[4\pi,6\pi]$, and use the periodicity of $\sin^2(\phi+2\pi) = \sin^2(\phi)$, to obtain

$$\frac{1}{3}\int_0^{6\pi} d\phi\delta(E - \tilde{E}_{\lambda,\xi}(k,\phi)) = \frac{1}{3}\sum_{\ell=0}^{2}\int_{2\pi\ell}^{2\pi(\ell+1)} d\phi\delta(E - \tilde{E}_{\lambda,\xi}(k,\phi)) = \int_0^{2\pi} d\phi\delta(E - \tilde{E}_{\lambda,\xi}(k,\phi)). \tag{S65}$$

Therefore, Eq.(S65) reduces to the expression

$$\begin{aligned} N_{sCDW}(E) &= \sum_{\lambda=\pm,\xi=\pm}\int_0^{2\pi}\frac{d\phi}{(2\pi)^2}\int_0^\infty dk\,k\delta(E - \tilde{E}_{\lambda,\xi}(k,\phi)) \\ &= \frac{1}{6\alpha^{2/3}}\sum_{\lambda=\pm,\xi=\pm}\int_0^{2\pi}\frac{d\phi}{(2\pi)^2}\int_{u^2+\Delta^2}^\infty \frac{d(\tilde{E}_0^2)}{\left(\tilde{E}_0^2 - (u^2+\Delta^2)\right)^{2/3}}\delta\left(E - \tilde{E}_{\lambda,\xi}(\tilde{E}_0^2,\phi)\right) \\ &= \frac{1}{6\alpha^{2/3}}\sum_{\lambda=\pm,\xi=\pm}\int_0^{2\pi}\frac{d\phi}{(2\pi)^2}\int_{u^2+\Delta^2}^\infty \frac{d(\tilde{E}_0^2)}{\left(\tilde{E}_0^2 - (u^2+\Delta^2)\right)^{2/3}}\sum_{\ell=\pm}\frac{\delta\left(\tilde{E}_0^2 - \epsilon_\ell(E,\phi)\right)}{\left|\frac{\partial\tilde{E}_{\lambda,\xi}}{\partial(\tilde{E}_0^2)}\right|}, \end{aligned} \tag{S66}$$

where in the second line we changed the integration variable $k \to \tilde{E}_0$ as defined in Eq.(S62). In the final step, we defined by $\epsilon_\pm$ the two independent roots of the equation $E - \tilde{E}_{\lambda,\xi}(\tilde{E}_0^2,\phi) = 0$, as follows

$$\epsilon_\pm(\phi) = E^2 + 2\Delta^2 \pm 2\Delta\sqrt{[E^2 - (u^2+\Delta^2)]\sin^2(\phi) + u^2 + \Delta^2\sin^4(\phi)}. \tag{S67}$$

In addition, the Jacobian in the denominator of Eq. (S66) is given by

$$\left.\frac{\partial \tilde{E}_{\lambda,\xi}}{\partial(\tilde{E}_0^2)}\right|_{\epsilon_\ell} = \frac{1+\frac{2\Delta^2\sin^2(\phi)}{E^2-\epsilon_\ell(\phi)}}{2E}. \tag{S68}$$

Finally, evaluating the integral and taking into account the support of the delta-function for each possible case, we obtain the expression

$$N_{sCDW}(E) = \frac{|E|}{3\alpha^{2/3}}\sum_{\ell=\pm}\int_0^{2\pi}\frac{d\phi}{(2\pi)^2}\Theta(\epsilon_\ell(\phi)-(u^2+\Delta^2))\left(\epsilon_\ell(\phi)-\left(u^2+\Delta^2\right)\right)^{-2/3}\left|1+\frac{2\Delta^2\sin^2(\phi)}{E^2-\epsilon_\ell(\phi)}\right|^{-1} \tag{S69}$$

Notice that at low-energies the DOS scales linearly with energy, consistent with the existence of the six non-degenerate band touching points in the Brillouin zone (see also Fig. 3 in the main text). This expression for the DOS is used for the plot in Figs. 2(c) in the main text.

3. *Optical conductivity*

We calculate optical conductivity in sCDW phase using the mean-field Hamiltonian, where we can set $\Delta_3 = 0$ and $\Delta_0 \equiv \Delta$. The collisionless optical conductivity will be considered at $T = 0$, $\mu = 0$, and $u = 0$, and for $\omega \gg \Delta$. We carry out the calculation following the steps outlined in Sec. (S2). First, we perform the trace in Eq. (S3). After performing transformations in the angular variable, ϕ, the integral over frequency and the substitution in the radial coordinate of the momentum, $y = \alpha k^3$, takes the form

$$\Pi_{xx}(i\Omega) = -i\frac{3}{4\pi}\sum_{\zeta=\pm}\int_0^{\pi/2} d\phi\int_0^\infty dy\; y\;\delta\left[g_\zeta(y)\right], \tag{S70}$$

where

$$g_\zeta(y) = \frac{\omega}{2} - \sqrt{(\Delta-\zeta y)^2 + 2\zeta\Delta y\left(1-\sin\frac{\phi}{2}\right)}. \tag{S71}$$

We now use the property of the $\delta-$function

$$\delta[f(y)] = \sum_i \frac{1}{|f'(y_{0i})|}\delta(y-y_{0,i}), \tag{S72}$$

where $f(y_{0,i}) = 0$, and the above sum is carried out over all the zeroes of the function $f(y)$. The zeroes of $g_\zeta(y)$ in Eq. (S71) are given by

$$y_{0,(\pm)}^{(\zeta)} = \zeta\Delta\sin\frac{\phi}{2} \pm \sqrt{\Delta^2\sin^2\frac{\phi}{2}+\frac{\Omega^2}{4}-\Delta^2}. \tag{S73}$$

In the universal limit $\Omega \gg \Delta$, the only two positive zeroes are

- $y_{0,(+)}^{(+)} = \Delta\sin\frac{\phi}{2} + \sqrt{\Delta^2\sin^2\frac{\phi}{2}+\frac{\Omega^2}{4}-\Delta^2}$,
- $y_{0,(+)}^{(-)} = -\Delta\sin\frac{\phi}{2} + \sqrt{\Delta^2\sin^2\frac{\phi}{2}+\frac{\Omega^2}{4}-\Delta^2}$.

Using Eq. (S73), we then obtain

$$\begin{aligned}\Pi_{xx}(i\Omega) &= -i\frac{3}{4\pi}\left(\frac{\Omega}{2}\right)\int_0^{\pi/2} d\phi\int_0^\infty dy\; y\;\left[\frac{\Omega^2}{4}-\Delta^2\cos^2\frac{\phi}{2}\right]^{-1/2}\left\{\delta\left(y-y_{0,(+)}^{(+)}\right)+\delta\left(y-y_{0,(+)}^{(-)}\right)\right\}\\ &= -i\frac{3}{8}\Omega.\end{aligned} \tag{S74}$$

The Eq. (S2) implies that the conductivity in the collisionless regime of the sCDW phase at the mean-field level is a constant

$$\sigma(\Omega) = \frac{3}{8}\frac{e^2}{\hbar} = \frac{3\pi}{4}\frac{e^2}{h}. \tag{S75}$$

This result is consistent with the splitting of the two nodal points at the two valleys, with the vorticity $\pm 3\pi$ into six simple band touching points, featuring two triplets of the vorticity equal to $\pm\pi$.

Finally, one can explicitly check that in the collision-dominated regime, for $\omega \ll \Delta$, the the polarization tensor component in Eq. (S70) is purely real, and therefore, after the analytical continuation yields the Drude peak $\sim \delta(\Omega/\Delta)$. This is analogous to case of the band touching for noninteracting Dirac electrons at a finite temperature yielding the Drude peak $\sim \delta(\Omega/T)$.

[1] M. Koshino and E. McCann, Phys. Rev. B **80**, 165409 (2009).
[2] F. Zhang, B. Sahu, H. Min, and A. H. MacDonald, Phys. Rev. B **82**, 035409 (2010).
[3] V. Cvetkovic and O. Vafek, arXiv:1210.4923 (2012).
[4] A. L. Szabó and B. Roy, Phys. Rev. B **105**, L081407 (2022).
